# Schizophrenia: A Pathogenetic Autoimmune Disease Caused by Viruses and Pathogens and Dependent on Genes

**DOI:** 10.4061/2011/128318

**Published:** 2011-05-26

**Authors:** C. J. Carter

**Affiliations:** Polygenic Pathways, 20 Upper Maze Hill, St Leonards-on-Sea, East Sussex, TN38 OLG, UK

## Abstract

Many genes have been implicated in schizophrenia as have viral prenatal or adult infections and toxoplasmosis or Lyme disease. Several autoantigens also target key pathology-related proteins. These factors are interrelated. Susceptibility genes encode for proteins homologous to those of the pathogens while the autoantigens are homologous to pathogens' proteins, suggesting that the risk-promoting effects of genes and risk factors are conditional upon each other, and dependent upon protein matching between pathogen and susceptibility gene products. Pathogens' proteins may act as dummy ligands, decoy receptors, or via interactome interference. Many such proteins are immunogenic suggesting that antibody mediated knockdown of multiple schizophrenia gene products could contribute to the disease, explaining the immune activation in the brain and lymphocytes in schizophrenia, and the preponderance of immune-related gene variants in the schizophrenia genome. Schizophrenia may thus be a “pathogenetic” autoimmune disorder, caused by pathogens, genes, and the immune system acting together, and perhaps preventable by pathogen elimination, or curable by the removal of culpable antibodies and antigens.

## 1. Introduction

Over 600 genes have been implicated in schizophrenia in association studies, supporting the contention that multiple genes of small effect contribute to this condition [1, 2] (see http://www.polygenicpathways.co.uk/schizgenesandfunc.htm for association references). These genes cluster together in clearly defined signalling networks related to the diverse subpathologies of schizophrenia [3–7]. Epistasis between genes within these same signalling networks markedly affects the degree of risk-promotion [8–10], in part, explaining the inconsistency in genetic association studies.

 Schizophrenia has also been associated with prenatal complications including maternal rubella (German measles) [11], influenza [12, 13], Varicella zoster (chicken pox) [14], Herpes (HSV-2) [15], common cold infection with fever [16], or poliovirus infection [17] while in childhood or adulthood, coxsackie virus infection (in neonates [18]) or Lyme disease (vectored by the Ixodes tick and Borrelia Burgdorferri) or Toxoplasmosis have been reported as risk factors [19, 20] (see Table 1). The human endogenous retrovirus, HERV-W, has also been implicated in schizophrenia [21]. A number of schizophrenia-related genes are implicated in the life cycles of these pathogens, suggesting an interplay between genes and risk factors [22].

Many schizophrenia genes relate to the immune network [5, 6, 22, 23]. Immune activation is also observed in the schizophrenic brain [24, 25] or in lymphocytes [26–29]. A number of autoantigens/autoantibodies to key schizophrenia-related proteins have also been reported. These include dopamine, serotonin, acetylcholine, and NMDA receptors; inter alia (Table 2). Maternal immune activation in animal models has also been shown to generate phenotypes relevant to schizophrenia in the offspring [30]. 

 As shown below, genes, risk factors, and immunity can be linked together forming a unifying pathway whose elements are interdependent. Dysfunction of this network which is conditional upon interactions between its three branches may be responsible for schizophrenia.

## 2. Methods

The human herpesvirus 2 genome (NC_001798) as well as those of the rhinovirus (NC_001490), rubella (NC_001545.1) and Varicella zoster (NC_001348.1) and HERV-W (NP_055405.3: env polyprotein) viruses, Borrelia Burgdorferri (NC_011728) and T. Gondii (NC_001799: Partial genome) were screened against the human proteome using the NCBI BLAST server and the Entrez query filter “schizophrenia”. The HERV-W, influenza, HSV-2 and rubella viruses were also screened unfiltered (Translated pathogen genome versus human proteins: BlastX) [31]. The BLAST algorithm detects overall homology between entire gene or protein sequences, and it is necessary to set parameters to low significance levels in order to detect short intraprotein consensus homology. The parameters used were: Expect 20,000, *E* value = 100,000; matrix PAM30. The original BLAST results are stocked at http://www.polygenicpathways.co.uk/blasts.htm. Information for all abbreviations is available at this site, provided by the NextBio highlighting service.

BLAST files were scanned by an online tag cloud generator producing tags sized according to gene word occurrences http://www.tagcloud-generator.com/generator.php#anker. Word occurrences were counted using a “Highlightall add-in” for Firefox https://addons.mozilla.org/en-US/firefox/addon/4240/. 

 Antigenicity (B-cell epitope prediction) was estimated using the BepiPred server http://www.cbs.dtu.dk/services/BepiPred/ [32] (Table 4).

Kegg pathway analysis [33] of 632 schizophrenia susceptibility gene candidates was performed using Kegg mapper http://www.genome.jp/kegg/tool/color_pathway.html. The results of this analysis are available at http://www.polygenicpathways.co.uk/keggszgenes.htm. Venn diagrams were constructed online at http://www.bioinformatics.org/gvenn/index.htm [34]. 

 Genes and risk factors with at least one positive association are included in this study. Although certain genes and risk factors are clearly more important than others, and problems of replication in both gene and risk factor studies abound, gene, gene, and gene/environment interactions may explain some of the heterogeneity. For example many schizophrenia-related genes are involved in the life cycle of T. Gondii, but may be irrelevant if this pathogen is not encountered. Similarly T. Gondii infection may have little effect is such gene variants are not present. Pathway analyses of genome wide association data, and previous studies, are showing that the risk-promoting effects of many genes in similar pathways are better predictors of risk, than when treating each gene in isolation (see Section 1). Although some of these factors may be false positives, many genes and risk factors may have a role to play in certain conditions, but the greater import of genes such as DISC1 or neuregulin is recognised.

## 3. Results

**Figure 1:**
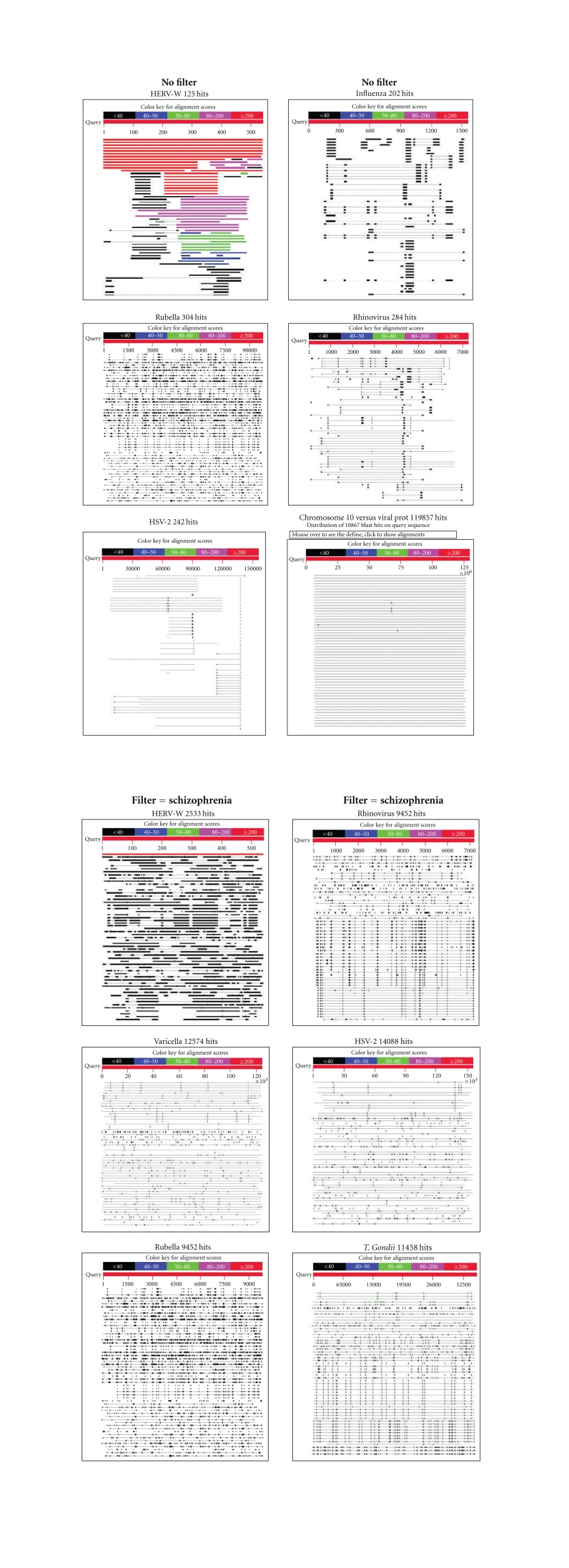
Screenshots of the pictorial representation of the viral BLAST results against the human proteome. The streaks dotted throughout the human genome/proteome represent the areas of homology, some with contiguous sequences of 5 or more amino acids. The number of hits is shown for each virus or pathogen. The figure also shows the total coverage of human chromosome 10 by viral gene homologues. The top set of figures were from unfiltered blasts while the bottom set of 6 figures represent filtered blasts using the query “schizophrenia”.

Pictograms of selected BLAST results are shown in Figure 1. The initial sweep of unfiltered BLASTs returned 125–304 hits, but this number was markedly increased when using the filter “schizophrenia” (14,088 Hits for HSV-2). For unfiltered sweeps, the viral homologues are longer, while the filtered sweeps return shorter contiguous sequences nevertheless including multiple matches of pentapeptides or more. 

**Figure 2:**
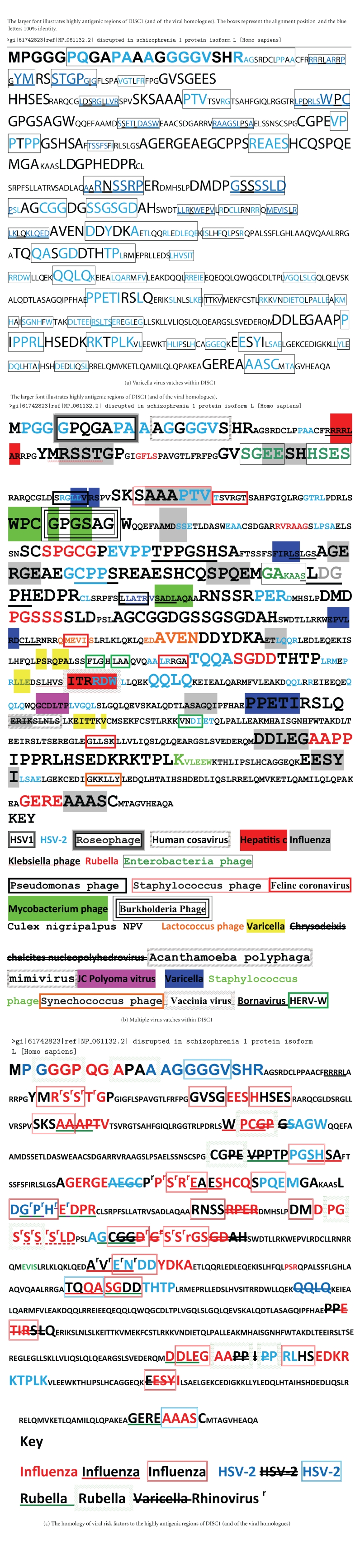
(a) Varicella protein alignments within DISC1: the boxed regions show the region of alignment, and the blue letters denote 100% identity. This is not an alignment of the whole Varicella proteome but represents fragments of the same or different Varicella proteins that align with DISC1 fragments (vatches). The larger font delineates highly antigenic regions of DISC1 with an antigenicity index of >0.8 (Figure 4). (b) Other viral vatches within the DISC1 protein. The vatches are colour or format coded in relation to the different viruses. (c) Viral vatches for the risk factors implicated in schizophrenia in relation to the highly immunogenic regions of DISC1.

**Figure 3:**
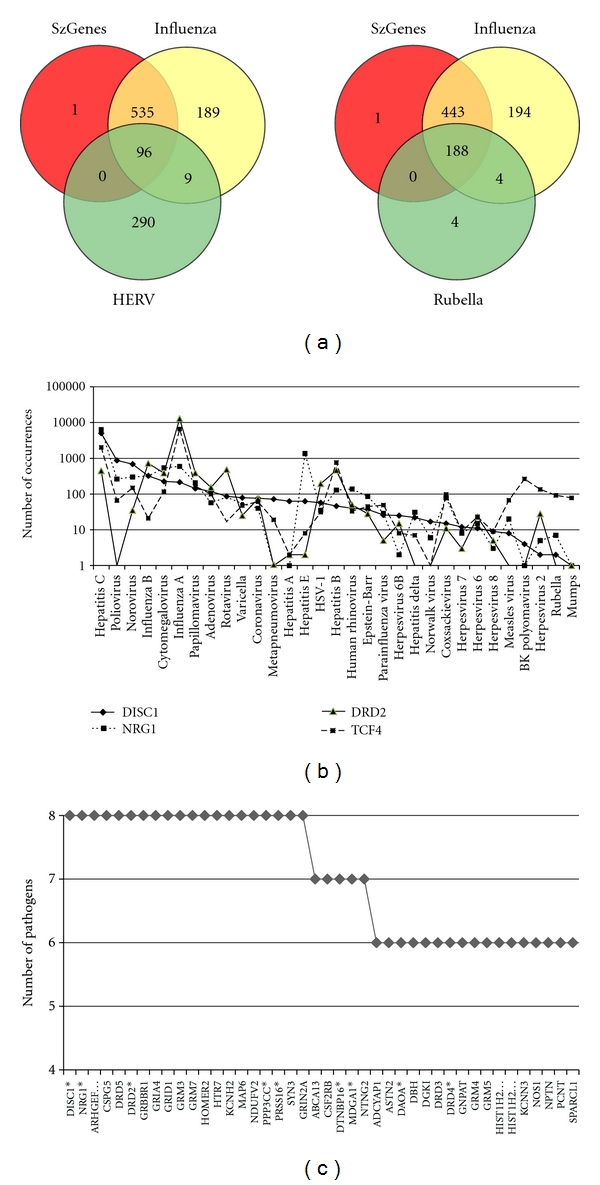
(a) Venn diagrams of the number of Schizophrenia gene products (*N* = 632) with homology to the rubella, HERV-W and influenza viruses. The singleton in SZ-genes was different on each occasion: Thus, all genes are covered. (b) The viral matching spectra of DISC1, neuregulin, the dopamine D2 receptor and transcription factor 4. The *Y*-axis depicts the number of word occurrences on the original BLAST results page. Note the logarithmic axis. (c) The number of pathogens expressing proteins with homology to the protein products of schizophrenia susceptibility genes. Those marked by an asterisk are within the 30 top-ranked genes in SZ-gene http://www.szgene.org/.

Viral-human matches are characterised by short contiguous amino acid matches of 5 or more amino acids, that are identical in viral and human proteins, defined as vatches (viral matches). These are exemplified, for DISC1 in Figure 2. Hexapeptide matches have also been described for the influenza H5N1 virus and this study also highlighted homologies with DISC1, reelin and neurexin, inter alia [35]. The entire length of a human protein can be composed of many overlapping, intercalated vatches, related to multiple viral species. However, the viral spectrum is distinct for each protein as shown in Figure 3 for DISC1, neuregulin, the D2 dopamine receptor and transcription factor 4. Each is homologous to proteins from a large spectrum of viruses, but this spectrum is distinct for each protein. Interestingly, all are homologous to proteins from the hepatitis C virus. Several studies have noted that Hepatitis C infection is associated with schizophrenia, but this has generally been interpreted in terms of a schizophrenia life style that favours infection, rather than viewing Hepatitis C as a risk-promoting factor [36–39]. These data may challenge this assumption. 

All of the pathogens implicated in schizophrenia express proteins with homology to multiple schizophrenia susceptibility gene products (Table 3). The profile of each individual pathogen is again specific for different types of gene product, but all target key members of the schizophrenia network including dopamine, serotonin and glutamate receptors as well as neuregulin and growth-related or DISC1 related pathways. This is the case even when no filter is used. Interestingly, both the rubella and the influenza viruses target members of the translation initiation complex, which has been implicated in myelination and oligodendrocyte survival [4, 40]. Oligodendrocyte cell loss and myelination defects are prominent in the schizophrenic brain [41–44]. 

The degree of overlap between the rubella, HERV and influenza viruses and schizophrenia gene products is shown by the Venn diagrams in Figure 3. All but one schizophrenia gene product was covered by various permutations and similar data were recovered for other pathogens. All schizophrenia gene products (*N* = 632) were homologous to proteins expressed by one or more of these pathogens. However, only 16 proteins were common to all 8 pathogens (Figure 3). These included neuregulin (NRG1) and DISC1, dopamine (DRD5), glutamate (GRIA4, GRID1, GRM3, GRM7) GABA (GABBR1) and serotonin (HTR7) receptors, a presynaptic protein regulating glutamate release (synapsin SYN3) and HOMER2, a member of the postsynaptic scaffold, all of which are key elements relating to the pathology of schizophrenia.

Other proteins within this class included neurocan (CSPG5), a chondroitin sulphate proteoglycan expressed in oligodendrocytes that inhibits neurite outgrowth and regulates axonal growth [45–47]. It is also involved thalamocortical projection development [48]. ARHGEF10 is a rho Guanine-nucleotide exchange factor that controls myelination [49]. NDUFV2 is a subunit of the mitochondrial respiratory chain and its protein expression levels are reduced in the frontal cortex and striatum in schizophrenia [50]. PPP3CC Calcineurin gamma (PPP3CC) plays a role in dopamine receptor signalling [51, 52]. Calcineurin knockout mice show defects in prepulse inhibition and other phenotypes related to schizophrenia [53]. Calcineurin is highly expressed in the immune system and regulates the expression of numerous cytokines [54]. MAP6 is a microtubule protein that controls synaptic organisation, in particular of glutamatergic synapses where it controls the expression of the glutamate transporter and presynaptic genes, synaptophysin and GAP-43, spinophilin and MAP2. [55, 56] KCNH2 is a potassium channel that plays a role in the development of neural crest cells [57] and in lymphocyte proliferation [58]. PRSS16 is a serine protease involved in autoimmunity and the presentation of self-antigens within the thymus [59]. 

 So, by a random bioinformatics process, trawling the entire human proteome, asking simply which proteins are homologous to those of the pathogens implicated in schizophrenia, we arrive at a small set of proteins related to synaptic and dendritic function, myelination, neuregulin and DISC1 pathways, glutamate, dopamine, GABA and serotonin transmission, and immune regulation that are the cornerstones of schizophrenia pathology [3, 60–62].

### 3.1. Autoantigens in Schizophrenia

Many autoantibodies have been reported in schizophrenia. The pathogens implicated in schizophrenia also express proteins that are homologous to these autoantigens. Again the profile of each autoantigen or pathogen is distinct as shown in Table 2. 

### 3.2. DISC1

DISC1 is a key “hub gene” in schizophrenia linked, via its interactome, to many other schizophrenia susceptibility gene products [3, 63–66]. Its viral homology is illustrated in Figure 2. The Varicella virus is homologous to DISC1 in several regions, over its entire length, many matches in regions of high immunogenicity. These figures illustrate the types of matches seen in other proteins and shows that the vatches are often part of larger gapped consensus sequences. Interestingly, Varicella infection also results in the production of antibodies to pericentrin, a DISC1 binding partner [67]. 

**Figure 4:**
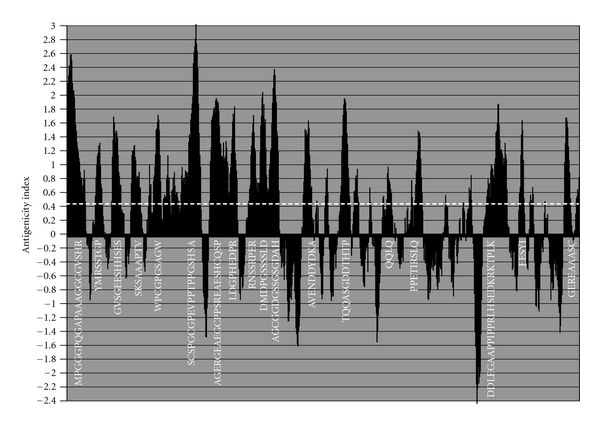
The antigenicity (B-cell epitope prediction) of DISC1: the amino acid sequences with an index of  >0.35 are considered as epitopes. A value of 0.8 was chosen to define highly antigenic regions as seen in Figure 2. The amino acid sequences of these highly antigenic regions are shown.

 DISC1 is a highly immunogenic protein, as predicted by B-cell epitope prediction (Figure 4). Autoantibodies to DISC1 have not been reported in schizophrenia. However, the viral risk factors implicated in schizophrenia express proteins that are homologous to the highly antigenic regions of the DISC1 protein, as shown in Figure 2. These viral proteins are equally antigenic and antiviral antibodies might also thus be expected to target multiple regions of the DISC1 protein.

### 3.3. Viral Proteins Are Part of the DISC1 Interactome

**Figure 5:**
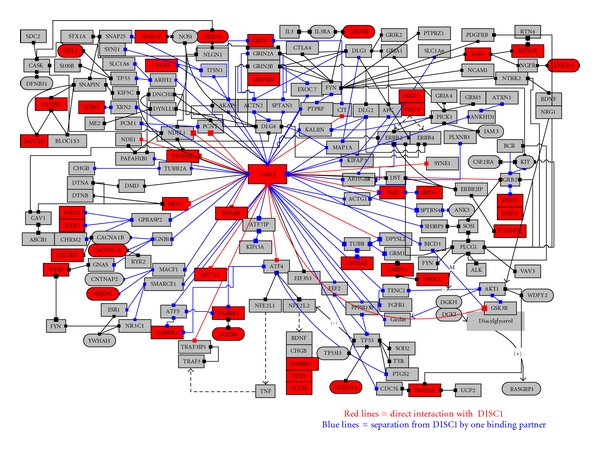
The DISC1 interactome see http://www.polygenicpathways.co.uk/discforum.htm. Proteins in red are homologous to Rubella proteins.

DISC1 and many of its binding partners, or other members of its interactome, contain vatches that are homologous to proteins expressed by the Rubella virus (Figure 5). (Other viruses also display this property, although the interactome members targeted are distinct, and specific for each virus (see http://www.polygenicpathways.co.uk/vatches.htm). Upon infection, viruses might therefore be considered as extraneous spurs to these types of protein/protein networks, and are likely to markedly affect their integrity. Indeed, several viruses, including herpes simplex, hepatitis C, Epstein-Barr, the cytomegalovirus, adenovirus and Coxsackie virus are known to bind to DISC1 interaction partners (Table 4).

### 3.4. Viral DNA within the Human Genome

The insertion of viral DNA into the human genome had until recently been thought to be the preserve of retroviruses. However the incorporation of DNA into mammalian genomes has recently been demonstrated on a large scale for both RNA and DNA viruses. Viral integration may be mediated by nonhomologous recombination with chromosomal DNA or, in the case of RNA viruses, by interactions with host chromosomal retrotransposons [68, 69]. It has also been shown the herpes virus HHV-6 can be transmitted from parent to child via chromosomal integration [70]. The BLAST analyses of the viruses detailed in this paper, and of others at http://www.polygenicpathways.co.uk/blasts.htm clearly show that viral DNA from many species is present within the human genome. This viral homology may well cover the entire human genome. For example, a Blast of human chromosome 10 against all viral genomes (almost 3,000 viral forms) yielded 119,857 hits with entire coverage of 135.5 million bases. Viral DNA is thus both inter and intragenic (Figure 1). It has been proposed that retroviral integration, into paternal and maternal gene lines, inserting several genes at once and effectively creating a new being, is responsible for evolutionary saccades [71]. The fact that RNA and DNA nonretroviruses can also be so incorporated has important implications in this area.

**Figure 6:**
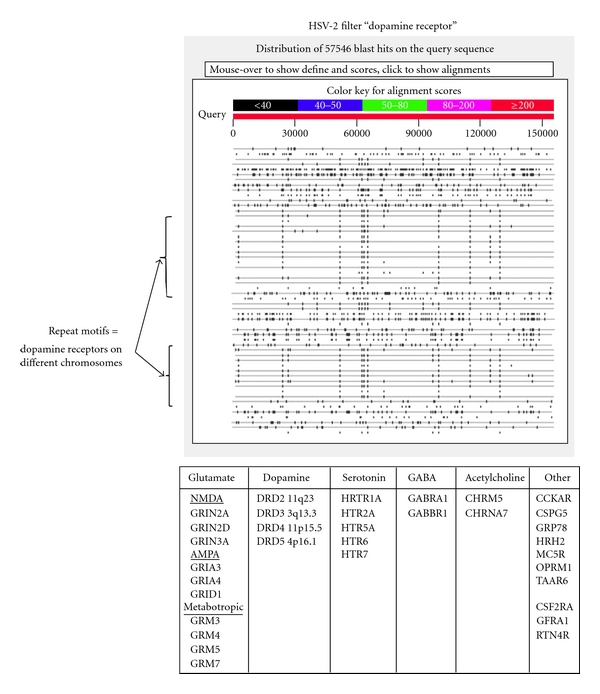
A screen shot of the HSV-2 BLAST results using the filter “dopamine receptor”. The repeated patterns correspond to dopamine receptors on different chromosomes as shown in Table 1. Homology with glutamate, serotonin, GABA, acetylcholine and other receptors is also noted.

The HSV-2 virus is homologous to several dopamine receptors and the BLAST pictogram shows how the same virus provokes repeating patterns in the human proteome (Figure 6). The same is true of the Herpes simplex virus (HSV-1) which is homologous to multiple lipoprotein receptors as well as to multiple kinases or of the cytomegalovirus which expresses proteins homologous to many chemokine receptors (see http://www.polygenicpathways.co.uk/blasts.htm). One interpretation of this, given the ability of chromosomal integration, is that repeated viral visits to the human genome over millions of years are responsible for the creation of gene families.

It is also possible that viral/human homology reflects convergent viral evolution, although this is difficult to reconcile with the presence of viral DNA in intergenic regions, for which there would be little evolutionary drive or selective pressure. It is also plausible that a bidirectional transfer of human and viral DNA could be at work. 

 For whatever reason, the result is that human proteins resemble those expressed by a multitude of today's viruses and other pathogens. Upon infection, these pathogens are thus able to interfere with the function of their human counterparts in a number of ways (see below).

### 3.5. Copy Number Variations and the Effects of Parental Age on Risk

Repeated viral insertion could well explain copy number variations, which are associated with a number of diseases, including schizophrenia [72, 73]. As their number increases, so will the number of matches to the same viral proteins, thus increasing the risk of viral interference and autoimmunity. As viral infection can be passed from parent to child via chromosomal integration, perhaps this is also why both paternal and maternal older age have been reported as risk factors in schizophrenia and other disorders [74, 75]. 

### 3.6. KEGG Pathway Analysis of Schizophrenia Susceptibility Genes

The color-coded pathways for this analysis are posted at http://www.polygenicpathways.co.uk/keggszgenes.htm. It confirmed the involvement of a number of polygenic pathways, including long-term potentiation and oxidative stress [3] growth factor/neuregulin pathways [121], neuroactive ligand pathways (dopamine/serotonin/glutamate and others) as well as dopamine metabolism pathways [9]. In the context of this review, a large number of immune-related pathways are traced out by these genes, together with many pathogen-related pathways, including toxoplasmosis, which heads the list (Table 5). The involvement of schizophrenia related genes in the life cycles of pathogens has been the subject of a previous review [22] and this relationship is supported by this analysis. Other pathogen related pathways relating to amoebiasis, Staphylococcus aureus and Helicobacter pylori infection, might indicate the involvement of other pathogens in schizophrenia, although such pathways could also be considered as generic pathways related to many pathogens. 

 There is no specific viral life cycle pathway within the KEGG dataset. However, viruses use adhesion molecules as receptors, endocytosis for cellular entry and the intracellular actin and tubulin networks for migration to and from the nucleus, mediated via dynein and kinesin motors. They also subjugate intracellular vesicular trafficking pathways, and are able to subvert both lysosomal and phagosomal pathways. Their exit may depend upon exocytosis, or by apoptotic or other means of killing their host cell [122]. These pathways are heavily represented within the schizophrenia gene analysis.

### 3.7. Mechanisms of Action

Individual proteins are homologous to multiple viral proteins, which nevertheless are specific for a spectrum of viruses, while individual viruses are homologous to a large but specific subset of human proteins. 

Our proteomes therefore contain proteins with sequences exactly matching those in the current virome, and in the proteomes of bacteria and other pathogens, which are also subject to phage or viral infection. Pathogens' proteins are therefore homologous to receptors, transporters, peptide messengers, growth factors, and other protein products of diverse gene families. Upon infection, surrogate dopamine, NMDA serotonin and other receptors, as well as transporters and enzymes are made available, which in effect may steal the ligands of their human counterparts. It is already known that the dopaminergic ligand, amantadine, binds to the influenza virus [123], which expresses proteins homologous to dopamine receptors (Table 3). When homologous to peptide ligands, viral proteins may occupy and block or perhaps stimulate their cognate receptors, or use them for entry, as is the case with the AIDS virus and the CCR5 and CXCR4 chemokine receptors [124]. 

This is illustrated by the Norovirus (Norwalk) which causes vomiting sickness. The virus expresses proteins homologous to monoamine and other amine oxidases as well as to a number of dopamine and monoamine transporters (Table 6). Dopamine subversion by the viral homologues would be expected to increase dopamine levels resulting in emesis, thus explaining the recurrent vomiting produced by infection.

The potential interference by viruses within protein/protein networks is well illustrated by the homology of rubella proteins to DISC1 and other members of its interactome, and by the fact that many viruses have indeed been found to bind to these components (Table 4).

 The homologous human proteins of the viral risk factors implicated in schizophrenia correspond to the genomic locations of 632 schizophrenia susceptibility genes (see Venn diagrams). Both negative and positive genetic association results have been reported for these many genes and it now seems plausible that, in some cases, this may be due to the presence or absence of active infection with these and other pathogens, and that DNA assays have been detecting pathogen as well as human DNA in the blood samples used for assay. There is evidently no way of discriminating viral or bacterial double-stranded DNA from human DNA.

This is not specific to schizophrenia, as the viruses implicated in Alzheimer's disease (HSV-1, HIV-1, HHV-6 and the cytomegalovirus) [125–127] are also homologous to proteins encoded by Alzheimer's disease susceptibility genes see http://www.polygenicpathways.co.uk/blasts.htm [128].

 It seems that a viable interpretation, given the same phenomenon in these diseases, is that these genes are susceptibility genes precisely because they encode for proteins with homology to the viral risk factors. Infection and genetics therefore appear to be interdependent. The pathogens may promote disease if the human genes encode for homologous products, and the genes promote disease if the homologous pathogen is encountered. Such interdependence likely explains the heterogeneous data in both gene and risk factor association studies. 

Other pathogens, including Borrelia Burgdorferri and T. Gondii have also been implicated in schizophrenia. These too express many homologous proteins to both viral and human proteomes. These parasites tend to be associated with schizophrenia in adulthood, while viral infections are predominantly prenatal risk factors. These may have primed the antibody network to respond to homologous antigens expressed by Borrelia or T. Gondii, suggesting that detection and elimination of these pathogens may be of therapeutic benefit in adult life. 

Schizophrenia is a neurodevelopmental disorder [129, 130] and, as the risk-promoting effects of viruses are related to maternal infection, it is possible that knockdown or interference of foetal proteins by viral-induced antibodies targeting their human counterparts may contribute to the neurodevelopmental disturbances observed in schizophrenia. Indeed DISC1, neuregulin, ERBB4, FEZ1 or COMT knockout mice display many of the pathological and behavioural symptoms associated with schizophrenia [131–135]. Viral interference with these same proteins might be expected to promote the same effects, but on a massive scale, targeting many relevant proteins at once. It is also possible that such autoantibodies play a role in the comorbid conditions associated with schizophrenia, for example autoimmune disease such as Thyrotoxicosis, celiac disease, acquired haemolytic anaemia, interstitial cystitis, or Sjogren's syndrome [136].

Autoantibodies to several proteins have been reported in schizophrenia (muscarinic, nicotinic, dopaminergic and NMDA receptors, *inter alia*, (Table 2) and all are homologous to proteins expressed by the risk factors in schizophrenia. The effects of antibody knockdown have not been analysed for any schizophrenia related proteins, but have been reported for the microtubule-related protein *tau*, in relation to Alzheimer's disease. In mice, *tau* immunisation produces *tau* hyperphosphorylation, neurofibrillary tangles and axonal damage as seen in the human condition [137]. *Tau* (MAPT) is homologous to Herpes simplex (HSV-1) and a number of other pathogens. Such effects are relevant to the autoantigens observed in schizophrenia.

 Schizophrenia is also a degenerative disease in adolescence or adulthood, characterised by oligodendrocyte cell loss, impaired synaptic connectivity and pyramidal cell dendrite shrinkage [41, 138–140], In the light of the above homologies it seems likely that such degenerative changes may relate to autoimmune-related attack of these diverse compartments. Indeed there is evidence for microglial activation in the schizophrenic brain [141] and several studies have reported changes in the cytokine profile in the brain, CSF or peripheral immune compartments [24, 142–146].

### 3.8. Clinical Implications in Schizophrenia and Other Conditions

These data suggest that susceptibility gene products are the vehicles enabling the risk-promoting effects of pathogenic risk factors, via the interactions described above, and that the two are indispensable for the genesis of schizophrenia. Pathogen detection and elimination or vaccination, particularly prior to pregnancy might be expected to reduce the incidence of schizophrenia and also to be of clinical benefit in adulthood. Interestingly, vitamin D is able to stunt the growth of T. Gondii [147] and low levels of this vitamin, both prenatally and in adulthood, have been associated with schizophrenia risk, although abnormally high levels are also a risk factor [148]. Pharmaceutical effort in this direction may also vastly improve the armoury and safety of drugs against parasites such as T. Gondii and Borrelia. 

Autoimmunity, involving several key schizophrenia-related proteins may well be a consequence of pathogen infection, and related to viral/human protein homology. Antigen and antibody removal by immunoadsorption techniques might therefore also be if clinical benefit. 

This scenario suggests a novel and probably common class of “pathogenetic” autoimmune disease caused by pathogens but dependent on our genes. Indeed, the same phenomenon has been observed in Alzheimer's disease where the risk factor herpes simplex expresses proteins containing peptide matches to the products of multiple susceptibility genes [128]. Work from Kanduc's laboratory has also shown that 30 viral proteomes, including many nonretroviruses, contain multiple pentapeptide matches to many human proteins [149]. This is corroborated by data posted at http://www.polygenicpathways.co.uk/blasts.htm which shows, *inter alia*, that Bornavirus proteins, a virus implicated in Bipolar disorder [150], display this type of homology in relation to Bipolar disorder susceptibility gene products, that the coronavirus implicated in Parkinson's disease [151] expresses proteins homologous to the PARK7 gene product and to dopaminergic and oxidative stress-related proteins, and that multiple sclerosis autoantigens are homologous to the products of the Epstein-Barr virus which has been implicated in this disorder [152]. Our genomes and polymorphisms determine which vatches we possess, which pathogens match these sequences and which pathogen-related disorder we might develop. Environmental variables, and vaccination, determine which pathogens we encounter and our immune system (HLA-antigens and immune background determined soon after birth) may determine how we deal with these pathogens. With the power of current day bioinformatics, it should be possible to rapidly identify all vatches in the human proteome and to pair them with the various pathogenic species and human diseases. This would greatly aid our understanding of the implication of pathogens in disease and may lead to radically new therapies and prevention strategies in many disorders.

## Figures and Tables

**Table 1:** Some of the pathogens implicated in Schizophrenia, either in relation to maternal infection, or to infection in later life.

Pre- and perinatal maternal infection	Juvenile (in offspring)	Adult
Rubella (first trimester) [76]: Influenza (first trimester) [13] Influenza or common cold with fever (second trimester) [16]	Mumps or cytomegalovirus infection (0–12 years old) [77]	HSV-1 seropositivity related to grey matter volume [78]

Poliovirus (second trimester) [17]	Coxsackie B5 infection perinatally [18]	HSV-1 (in Afro-Americans) or HHV-6 seropositivity: Inverse correlation with HSV-2 and cytomegalovirus [79]

Measles, Varicella zoster or polio (seropositivity at birth) [14]	Childhood meningitis (0–4 years old) [80]	Borna disease virus seropositivty [81]

HSV-2 (antibodies assayed at the end of pregnancy) [82]		Coronavirus seropositivity [83]

Influenza B (seropositivity at birth) [84]		Elevated retrovirus HERV-W transcripts [85]

Toxoplasmosis (antibodies during pregnancy) [86]		Measles virus seropositivity [87]

		Hepatitis C [38]

		Toxoplasmosis [88]

		Correlation with the incidence of Lyme disease (Borrelia) [20]

**Table 2:** Pathogens expressing proteins with homology to the autoantigens reported in schizophrenia. The size of the tags is proportional the number of pathogen's proteins that are homologous to the autoantigen. Note that the profile is different for each pathogen. The original BLAST files can be found at http://www.polygenicpathways.co.uk/blasts.htm.

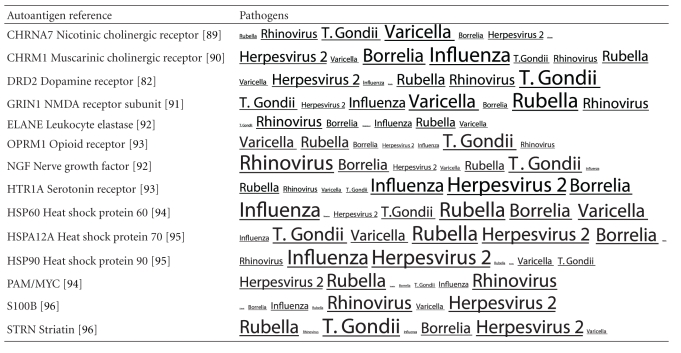

**Table 3:** Human proteins with homology to proteins expressed by pathogens. The size of the tags reflects the number of pathogen's proteins that are homologous to the human protein: the filters used are described. The number of schizophrenia susceptibility genes within each of these datasets is shown in the left-hand column. Certain genes are classified according to family and are highlighted in red. Gene definitions and the original BLAST files can be found at http://www.polygenicpathways.co.uk/blasts.htm. Note that the homologues are often clustered in families (e.g., HTR1A, HTR2A, HTR3A, HTR3B, HTR3E, HTR5A, and HTR7).

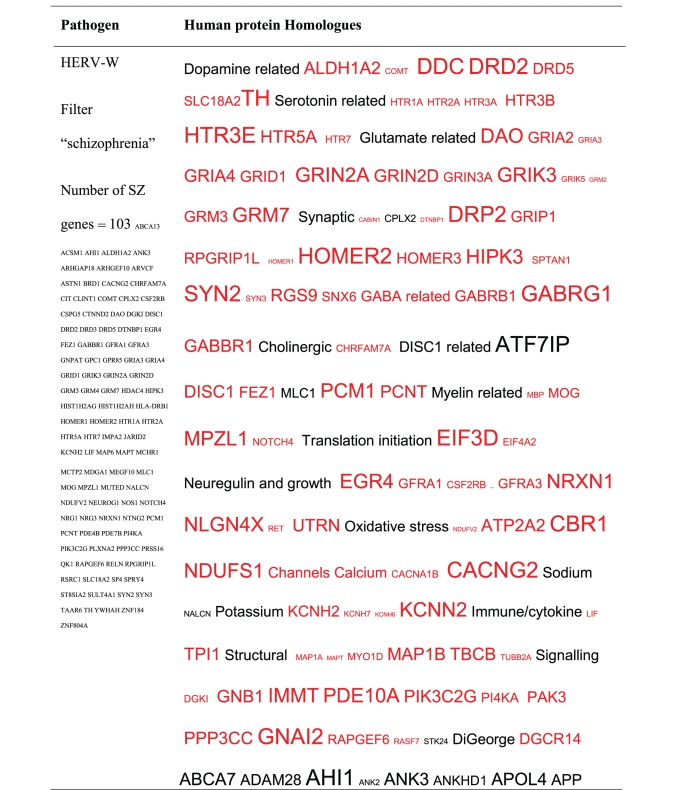 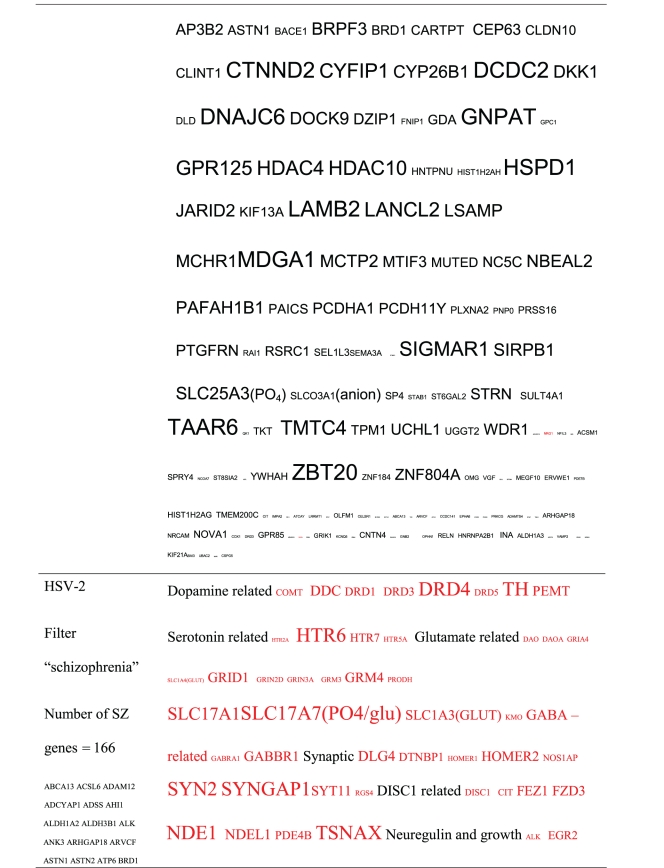 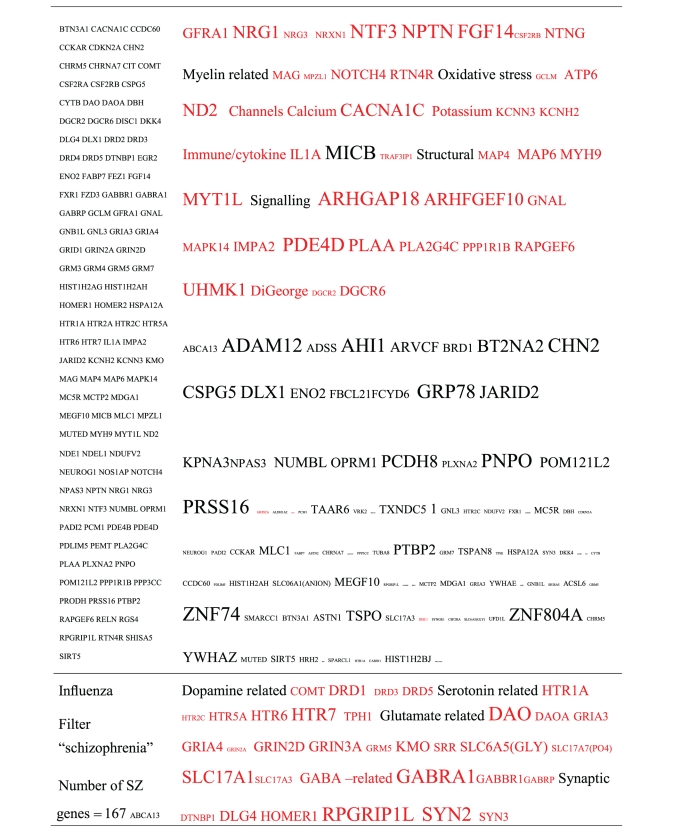 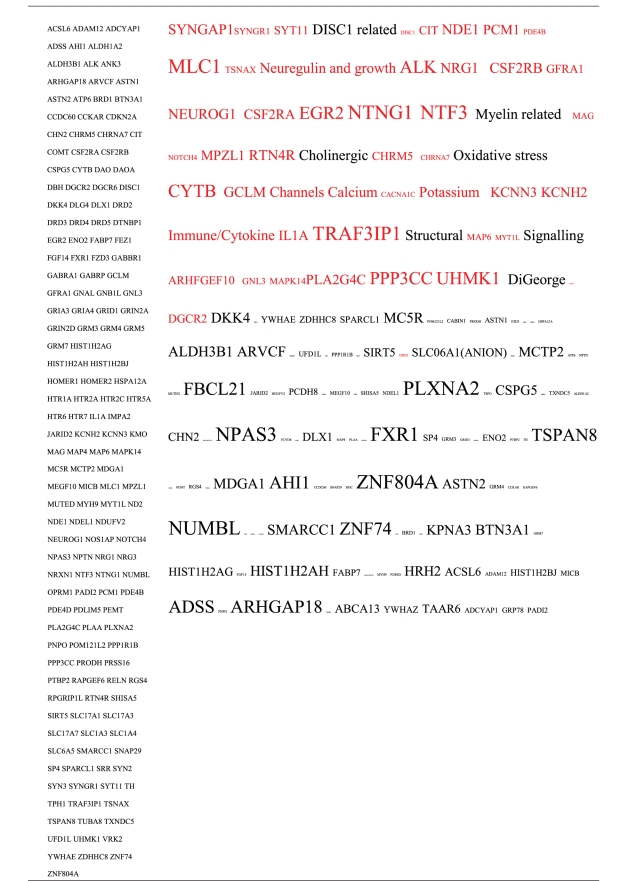 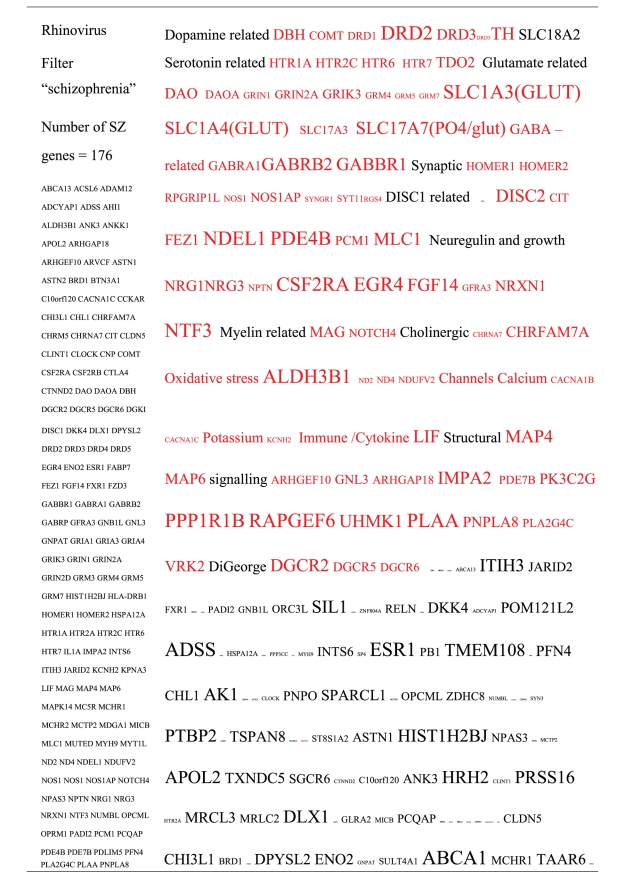 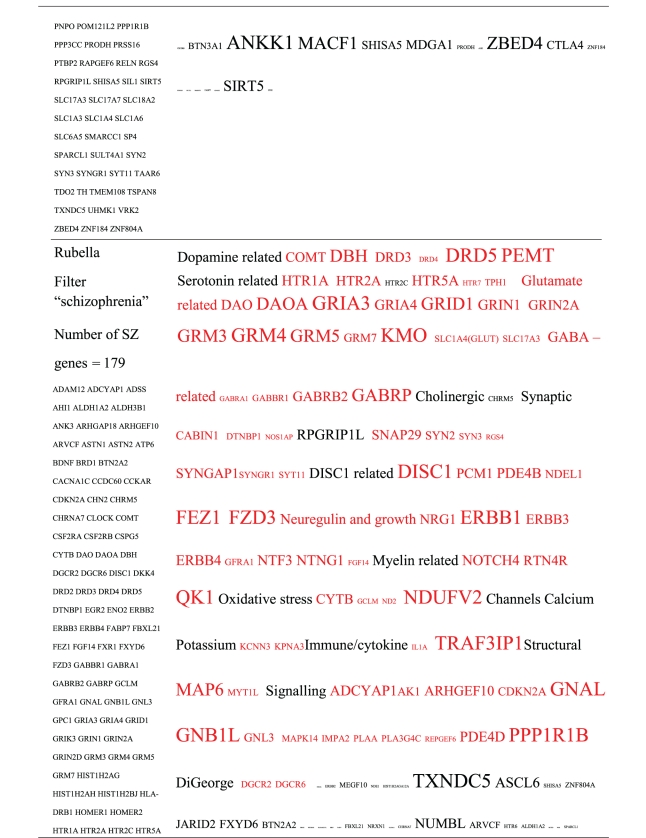 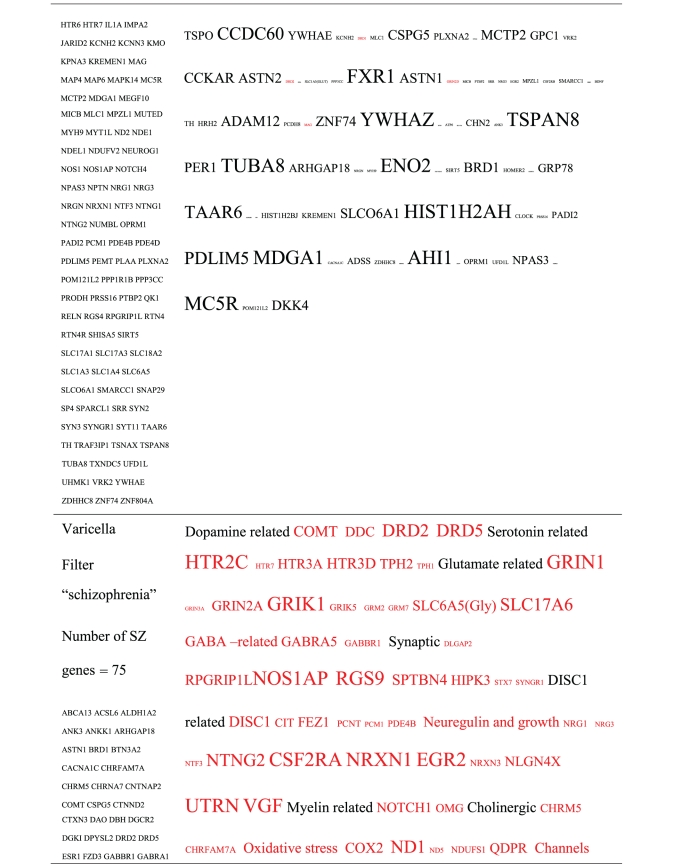 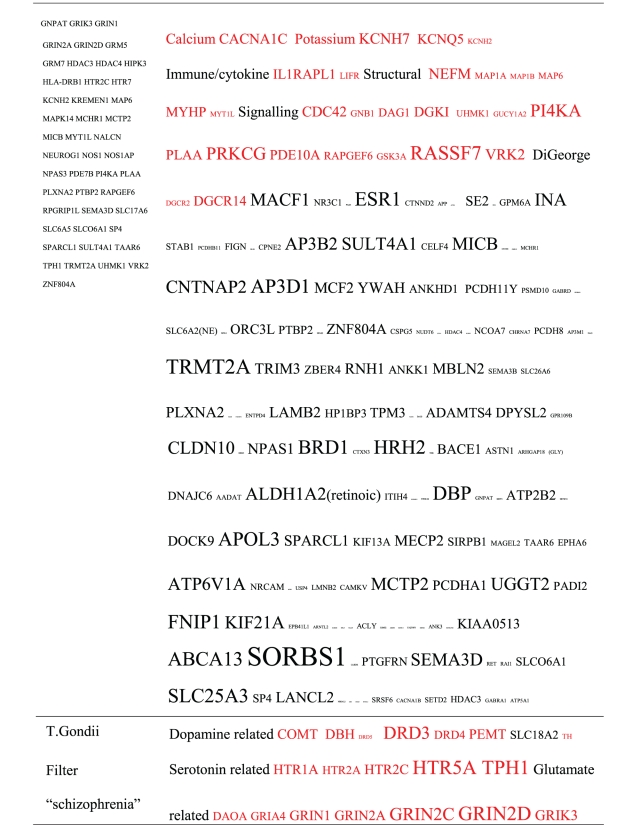 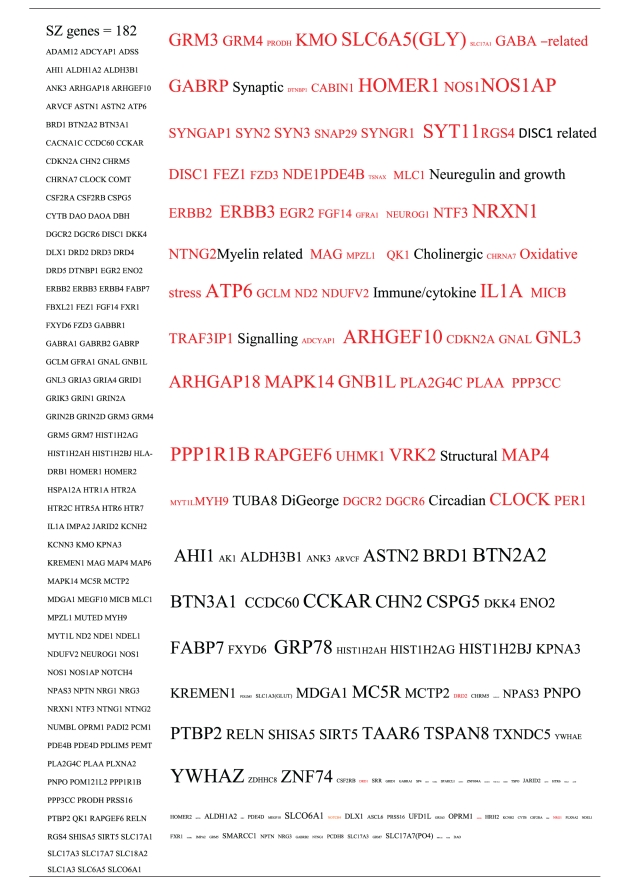 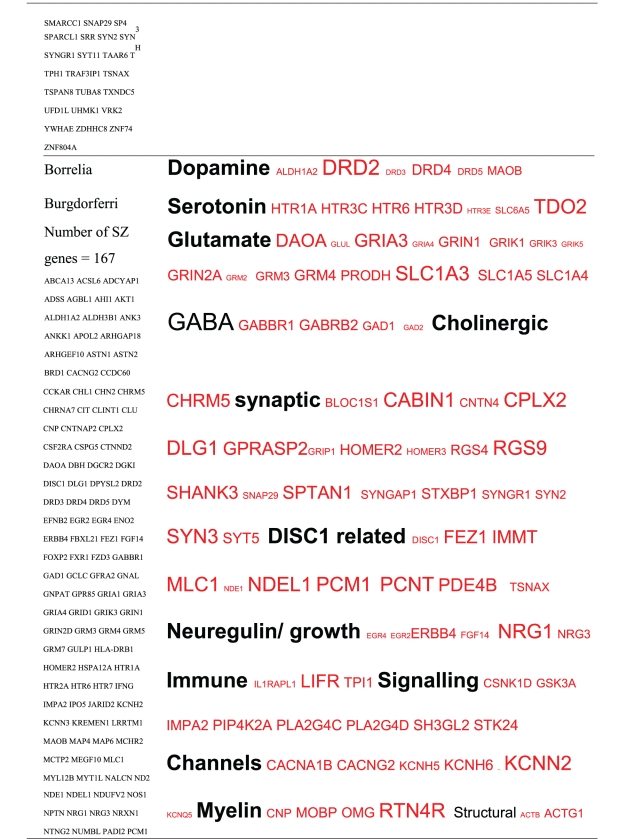 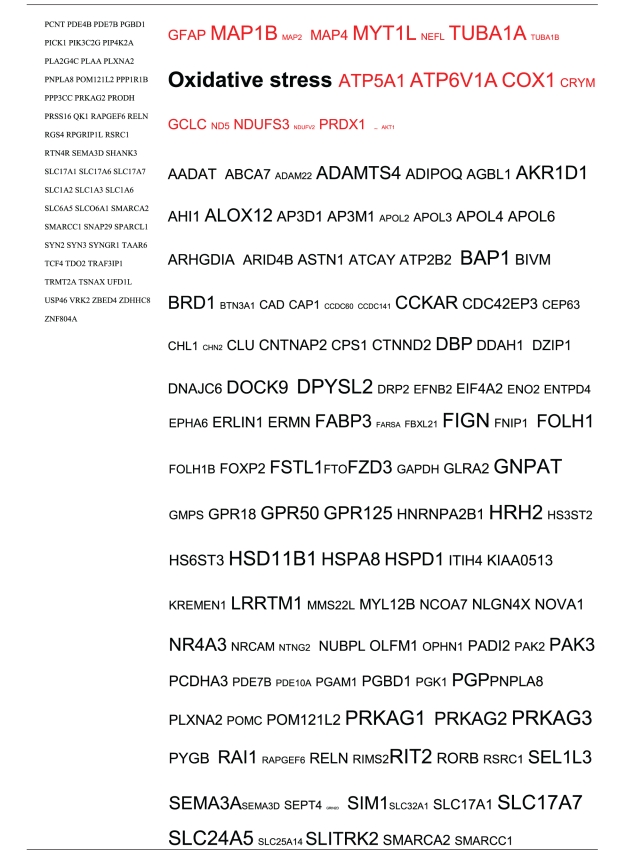 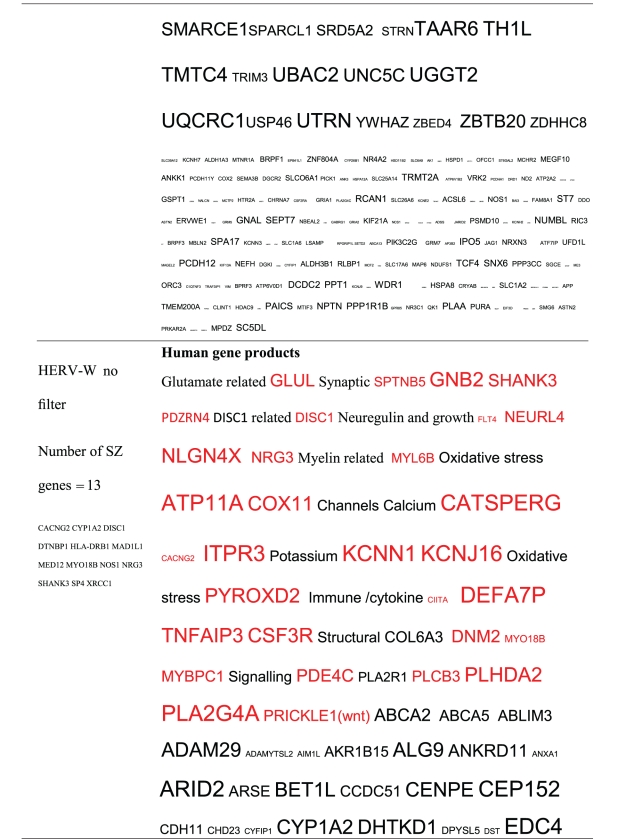 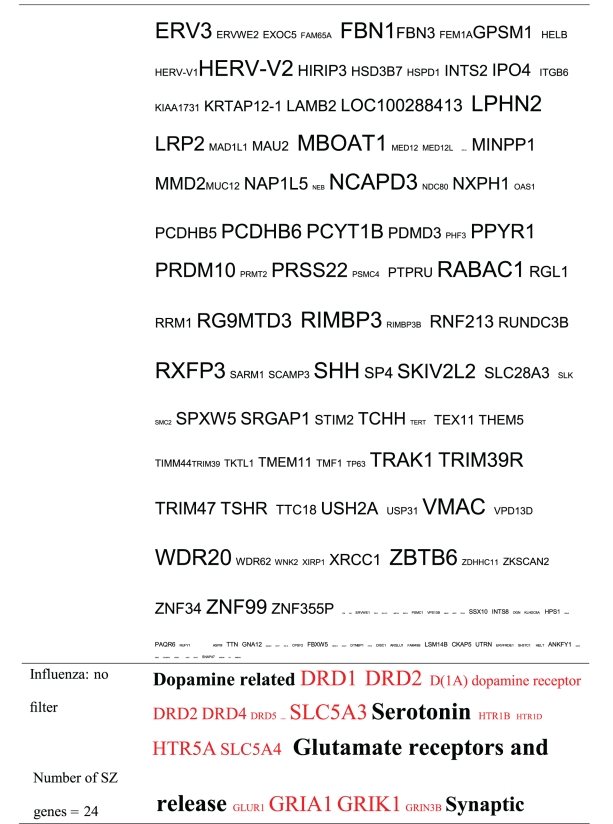 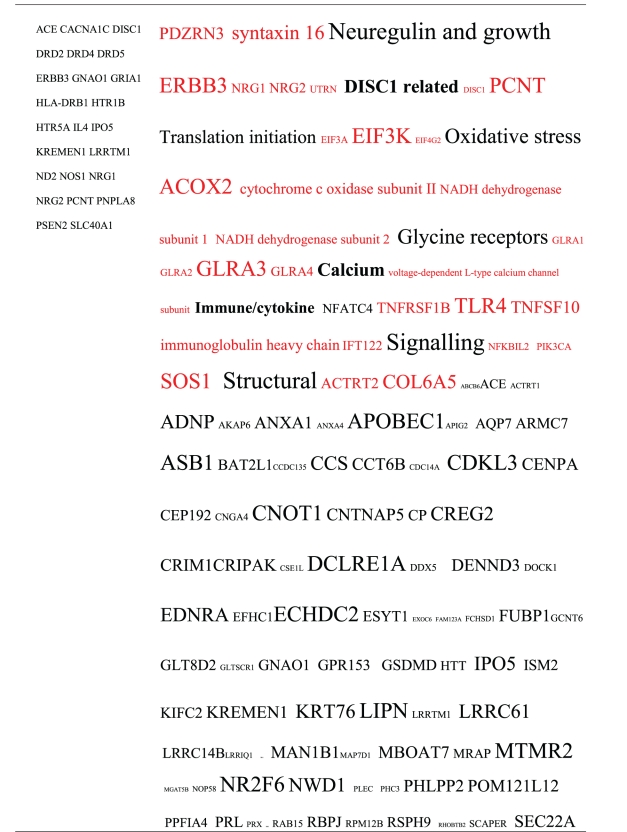 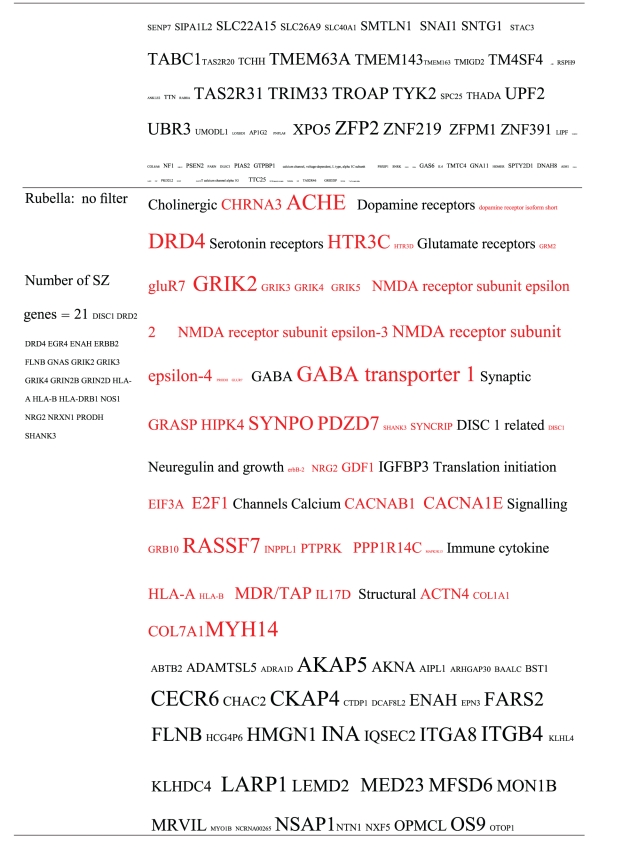 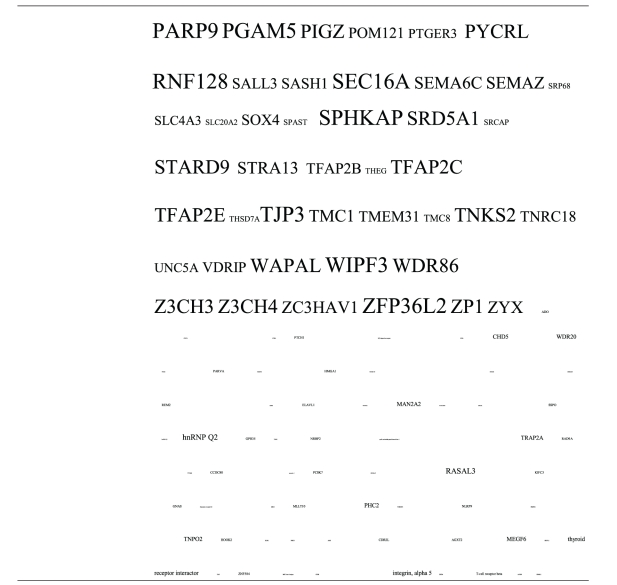

**Table 4:** Viruses reported to bind to DISC1 interactome partners.

DISC1 partner gene symbol	Protein name	Viral binder
ACTG1	Actin, cytoplasmic 2	HIV-1 [97] HSV1 [98]
ACTN2	Actinin, alpha 2	Hepatitis C [99]
AKAP9	A-kinase anchor protein 9	Epstein-Barr [97]
ATF4	Cyclic AMP-dependent transcription factor ATF-4	HSV1 [98]
ATF5	Cyclic AMP-dependent transcription factor ATF-5	HTLV1 [100]
BICD1	Protein bicaudal D homolog 1	Cytomegalovirus [101]
C14orf135	Uncharacterized protein C14orf135 precursor	Hepatitis C [102]
DCTN1	Dynactin-1	HSV1 [98]
DCTN2	Dynactin subunit 2	Dynactins are involved in the transport of the adenoviruses, HSV-1, the hantaan virus, HTLV-1 and the poliovirus [103–108]
DNAJC7	DnaJ homolog subfamily C member 7	Part of a complex forming the coxsackie virus receptor [109]
DYNC1H1	Dynein heavy chain, cytosolic	Adenovirus (in a complex with dynactin and NDEL1) [110]
EEF2	Elongation factor 2	Epstein Barr [111]
EIF3S3	Eukaryotic translation initiation factor 3 subunit 3	Hepatitis C [112]
FEZ1	Fasciculation and elongation protein zeta 1 (zygin I)	JC Polyomavirus [113]
HERC2	HECT domain and RCC1-like domain-containing protein 2	Papillomavirus 16 [114]
KIF3C	Kinesin-like protein KIF3C	HIV-1 [115]
MATR3	Matrin-3	HSV1 [98]
NDEL1	Nuclear distribution protein nudE-like 1	Part of a complex involved in Adenovirus transport (with dynactin and cytoplasmic dynein) [110]
PAFAH1B1	Platelet-activating factor acetylhydrolase IB subunit alpha	Binds to Poliovirus P3 protein and HIV-1 Tat [116, 117]
PCNT	Pericentrin	Involved in the microtubular transport of the adenovirus [118]
PGK1	Phosphoglycerate kinase 1	Epstein-Barr [119]
SMARCE1	SWI/SNF-related matrix-associated actin-dependent regulator of chromatin subfamily E member 1	HSV-1 [97]
STX18	Syntaxin-18	Papillomavirus [119]
TNKS	Tankyrase-1	Epstein-Barr [120]
TUBB	Tubulin beta chain	Epstein-Barr [119]
YWHAE	14-3-3 protein epsilon	Hepatitis C [97] : L : Epstein-Barr [119]
YWHAQ	14-3-3 protein theta	HIV [97] HSV1 [98]
YWHAZ	14-3-3 protein zeta/delta	HSV1 [98] : Epstein-Barr [119]

**Table 5:** The number of schizophrenia gene products in KEGG pathways related to immunity, and viral or pathogen life cycles.

Pathogen pathways		Viral pathways		Immune	
Toxoplasmosis	16	Focal adhesion	20	Cytokine-cytokine receptor interaction	26
Chagas disease	15	Cell adhesion molecules (CAMs)	19	Jak-STAT signaling pathway	16
Amoebiasis	13	Regulation of actin cytoskeleton	17	Systemic lupus erythematosus	13
Leishmaniasis	12	Protein processing in endoplasmic reticulum	13	T cell receptor signaling pathway	13
Viral myocarditis	8	Endocytosis	12	Phagosome	12
Staphylococcus aureus infection	7	Phagosome	12	Allograft rejection	11
Epithelial cell signaling in Helicobacter pylori infection	6	Gap junction	11	Hematopoietic cell lineage	11
Malaria	6	Tight junction	11	Antigen processing and presentation	10
Tryptophan metabolism	6	Adherens junction	6	Fc epsilon RI signaling pathway	10
NOD-like receptor signaling pathway	4	ECM-receptor interaction	6	Apoptosis	10
Vibrio cholerae infection	4	Oocyte meiosis	5	Graft-versus-host disease	9
Bacterial invasion of epithelial cells	3	SNARE interactions in vesicular transport	4	Autoimmune thyroid disease	8
E.coli infection	3			Chemokine signaling pathway	8
RIG-I-like receptor signaling pathway	3	Basal transcription factors	3	Leukocyte transendothelial migration	8
Cytosolic DNA-sensing pathway	2	Spliceosome	2	Natural killer cell mediated cytotoxicity	8
Shigellosis	2	Aminoacyl-tRNA biosynthesis	1	Adipocytokine signaling pathway	7
		Base excision repair	1	Asthma	7
		RNA degradation	1	Intestinal IgA production	5
				Toll-like receptor signaling pathway	5
				Complement and coagulation cascades	4
				B cell receptor signaling pathway	3
				TGF-beta signaling pathway	3
				Lysosome	2
				Regulation of autophagy	2
				Fc gamma R-mediated phagocytosis	1
				Primary immunodeficiency	1

**Table 6:** Human homologues of Norwalk virus proteins.

Dopamine metabolisers	Amine transporters	Others
**AOC2 **amine oxidases **AOC3“” ** **KDM1A **amine oxidase demethylase **KDM1B“”** **MAOA **monoamine oxidase **MAOB“”** **RNLS **renalase amine oxidase **SMOX **spermine oxidase **SPR **sepiapterin reductase Monoamine synthesis cofactor **SULT1A1 **sulphotransferases **SULT1A3 **monoamine metabolite sulphation **SULT1A4 **	**SLC6A2 (Noradrenaline) ** **SLC6A3 (Dopamine)** **SLC18A1vesicular monoamine** **SLC18A2“”** **SLC22A2 organic cation** **SLC22A3 extraneuronal monoamine ** **SLC29A4** (Na^+^/H^+^)	**CADPS2 amine release activator** **CDCA7 cell division cycle associated 7** **CDCA7L ** **IL4I1 cytokine** **PICK1** postsynaptic scaffold
